# The Effectiveness of Teleglaucoma versus In-Patient Examination for Glaucoma Screening: A Systematic Review and Meta-Analysis

**DOI:** 10.1371/journal.pone.0113779

**Published:** 2014-12-05

**Authors:** Sera-Melisa Thomas, Maya Jeyaraman, William G. Hodge, Cindy Hutnik, John Costella, Monali S. Malvankar-Mehta

**Affiliations:** 1 Department of Epidemiology and Biostatistics, Schulich School of Medicine and Dentistry, Western University, London, Canada; 2 Department of Ophthalmology, Ivey Eye Institute, St. Joseph's Health Care London, London, Canada; 3 Allyn and Betty Taylor Library, Western University, London, Canada; Casey Eye Institute, United States of America

## Abstract

**Background:**

Glaucoma is the leading cause of irreversible visual impairment in the world affecting 60.5 million people worldwide in 2010, which is expected to increase to approximately 79.6 million by 2020. Therefore, glaucoma screening is important to detect, diagnose, and treat patients at the earlier stages to prevent disease progression and vision loss. Teleglaucoma uses stereoscopic digital imaging to take ocular images, which are transmitted electronically to an ocular specialist. The purpose is to synthesize literature to evaluate teleglaucoma, its diagnostic accuracy, healthcare system benefits, and cost-effectiveness.

**Methods:**

A systematic search was conducted to help locate published and unpublished studies. Studies which evaluate teleglaucoma as a screening device for glaucoma were included. A meta-analysis was conducted to provide estimates of diagnostic accuracy, diagnostic odds ratio, and the relative percentage of glaucoma cases detected. The improvements to healthcare service quality and cost data were assessed.

**Results:**

Of 11237 studies reviewed, 45 were included. Our results indicated that, teleglaucoma is more specific and less sensitive than in-person examination. The pooled estimates of sensitivity was 0.832 [95% CI 0.770, 0.881] and specificity was 0.790 [95% CI 0.668, 0.876]. The relative odds of a positive screen test in glaucoma cases are 18.7 times more likely than a negative screen test in a non-glaucoma cases. Additionally, the mean cost for every case of glaucoma detected was $1098.67 US and of teleglaucoma per patient screened was $922.77 US.

**Conclusion:**

Teleglaucoma can accurately discriminate between screen test results with greater odds for positive cases. It detects more cases of glaucoma than in-person examination. Both patients and the healthcare systems benefit from early detection, reduction in wait and travel times, increased specialist referral rates, and cost savings. Teleglaucoma is an effective screening tool for glaucoma specifically for remote and under-services communities.

## Introduction

Vision impairment represents a serious public health concern since it impacts social, mental, and physical health of an individual. Visual impairment limits independence and activities of daily life. Those with visual impairment require more social support systems, visual aids, and modifications to home life. They are at a higher risk for injuries, falls, psychological conditions, and are admitted to nursing homes earlier compared to those without a vision impairment [1].

Glaucoma is the leading cause of irreversible visual impairment in the world affecting 60.5 million people worldwide in 2010 [2]. In developed countries, half of glaucoma patients may not experience vision loss until the advanced stages of the disease and this is expected to be greater in undeveloped countries [3]. Since there is no cure for glaucoma, glaucoma can progress to blindness if left untreated. Further, glaucoma accounts for 12% of blind persons worldwide which is expected to increase to approximately 79.6 million in 2020 [4]. The largest impact is expected in China and India, which accounts for 40% of all cases together [4].

The burden of the glaucoma has affected both the health care and economic systems. In Canada, alone, vision loss costs the economy $15.8 billion per year in which 55% is allocated to direct health care costs [5]. Sixty-five per cent of adults with partial or full vision loss are unemployed, which translates to $4.06 billion annually of lost earnings [5]. The direct costs of glaucoma is estimated in the United States to be $623 for mild, $1915 for moderate, and $2511 for severe forms of glaucoma and similarly in Europe the costs are €455 per person each year for mild glaucoma and €969 per person each year for severe glaucoma [4]. Varma et al. reported as glaucoma progresses to each stage, there is an €86 increase in treatments costs in European.

Glaucoma screening is important to detect, diagnose, and treat patients at the earlier stages. Screening and diagnostic tools are significant to prevent glaucoma from progressing to advanced stages and maintaining health vision. In addition, glaucoma prevention will minimize future healthcare costs. Screening improves efficiency of the health care system by increasing the number of patients accessing ophthalmic services and it reduces the number of false-positive referrals to ophthalmologists [6].

The standard of care for glaucoma screening is routine optometrist visits every 2–3 years and any suspect glaucoma patient will be referred to an ophthalmologist for additional diagnostic testing [5]. Those of older ages are at a greater risk of glaucoma and thus ophthalmologists recommend routine optometrist visits every 2 to 4 years for adults between 40 to 64 years and every 1 to 2 years when aged 65 and older [7]. Patients regularly seen by ophthalmologist for other ocular conditions may also be referred for glaucoma diagnostic testing if signs appear. In-patient care for glaucoma (passive “in-person screening”) is performed at specialized clinics and includes detailed history, slit lamp examination, visual field testing, and fundus photography performed by the optical technician followed by consultation with the ophthalmologist [8].

Teleglaucoma is a relatively new screening and diagnostic tool for targeting remote or under-serviced communities. It uses stereoscopic digital imaging to take ocular images which are transmitted electronically to an ocular specialist. The ocular specialist will then assess the images, identify risk factors and diagnose for glaucoma. If necessary the ocular specialist will refer identified glaucoma cases for medical consultations or to ophthalmologists for follow-up treatment. Unlike other teleophthalmology tools, teleglaucoma requires more sophisticated diagnostic tests. The main tests are optic nerve photographs, Optical Coherence Tomography (OCT), Intraocular Pressure (IOP) measurements, central corneal thickness (CCT) measurements, and visual field tests [9]. The combination of examinations and equipment required can vary based on organizational resources, target goals and populations. However, the more diagnostic tools used during screening for glaucoma the greater the accuracy and effectiveness of the screening process. The equipment required for teleglaucoma are the ophthalmic examination equipment, cameras, and computer imaging software. The A full list of the standard equipment and components of teleglaucoma can be found in Table 1 [9].

**Table 1:** Standardized teleglaucoma equipment.

Components	Requirements
Human Resources	Staff: graders, Ophthalmic technicians, nurses, optometrist, physicians, glaucoma specialists/ophthalmologists
Information Technology	Secure Diagnostic Imaging (SDI) system
	Videoconferencing equipment
	Computer systems and software
	ISDN installation
Screening Equipment	Retinal camera
	Tonometer
	Devices to measure central corneal thickness
	Frequency Doubling Technology (FDT) or Humphrey Visual Field test
	Optical Coherence Tomography
	Slit lamp
	Gonioscope
	Retinal camera
	Tonometer
	Devices to measure central corneal thickness
Examinations	Medical & family history
	Visual acuity
	IOP
	CCT
	Pupil equal and reactive to light (PERL) or relative afferent pupillary defect (RAPD)
	Slit lamp
	Gonioscopy
	Visual field
	Fundus photographs
	OCT
	Ancillary tests

The advantages reported in the literature include convenience, decreased travel time to medical clinics, increased access to specialized care for glaucoma, and decreased patient costs [10], [11] The benefits are mainly seen in remote or under-serviced communities such as Aboriginal communities and rural or remote areas where there is limited ocular specialists. Arora et al. reported improved **access time** (time from patient being referred to the date visit is booked) with teleglaucoma versus standard in-person examinations: 45 days for teleglaucoma versus in-person exam which had 88 days [12]. Teleglaucoma had reduced **cycle time** (time from registration until patient leaves clinic) of 78 minutes versus in-person exam of 115 minutes [12]. The pioneer teleglaucoma study conducted in Finland reported reduced absence from work by 50% with teleglaucoma versus in-person examination, and in addition reduced traveling (97%), costs (92%), and time (92%) [11].

The literature suggests teleglaucoma has comparable diagnostic accuracy. Teleglaucoma technology demonstrated moderate agreement in its ability to diagnose glaucoma (Kappa statistic 0.55% (0.48, 0.62)) [13]. When disc damage had Vertical Cup Disc Ratio (VCDR) greater than 0.7 the Frequency Doubling Technology (FDT) had a substantial agreement with ability to diagnose glaucoma (kappa statistic 0.84) [13]. In addition, a study conducted in rural India compared the ability of teleglaucoma to detect glaucoma compared to standard in-clinic examination and found that there was good agreement in detecting glaucoma [14]. For glaucoma the kappa scores were 0.61 with standard screening versus 0.59 for teleglaucoma [14]. In comparison to the in-person slit lamp examination, the positive predictive value was 77.5% for positive teleglaucoma diagnosis and had a negative predictive value of 82.2% for negative teleglaucoma diagnosis [13]. However, a cohort study conducted by the University of Alberta found 24% of teleglaucoma photographs were deemed unreadable from media opacities, patient cooperation, and unsatisfactory photographic techniques [13].

In this study, a systematic review and meta-analysis was conducted on teleglaucoma screening for patients with glaucoma to evaluate the following: the effectiveness of teleglaucoma as a screening device, its diagnostic accuracy, its diagnostic odds ratio, and its cost-effectiveness in comparison to in-person examination. Section 2 will explain the methods, section 3 provides the detailed analysis, and section 4 concludes with a discussion and implications for future research.

## Methods

### Search Strategy

A search methodology was used to assist in locating both published and unpublished studies. Research databases and conference meeting abstracts were searched for articles published from 1999 to current, and included MEDLINE (OVID and PubMed), Cochrane Library (Wiley), BIOSIS (Thomson-Reuters), CINAHL (EBSCO), Web of Science (Thomson-Reuters), and EMBASE (OVID). The grey literature was explored by searching Dissertations and Theses (ProQuest), the Canadian Health Research Collection (Ebrary), as well as the annual meeting abstracts of the European Society of Ophthalmology, Canadian Society of Ophthalmology (CSO), Association for Research in Vision and Ophthalmology (ARVO), and American Academy of Ophthalmology (AAO). The Conference Proceedings Citation Index was also included as part of the Web of Science search. Hand searches of ARVO's *Investigative Ophthalmology & Visual Science* journal and *Canadian Journal of Ophthalmology* associated with CSO were performed. The search strategies employed database specific subject headings and keywords for glaucoma, tele-screening, detection, and their synonyms. Each strategy was structured to accommodate for database and platform specific terminology, and syntax. Supplementary File S1 contains the complete search strategies used for the various databases (Table S1). Alerts were set up for each database to receive publication notifications for new related articles.

### Inclusion and exclusion criteria

Articles included were from any country, all in English, published from 1999 to current, and were research articles. The articles included study population that are adults in the general population or populations at risk of glaucoma. The study population included those with or without glaucoma. Articles on teleglaucoma intervention for glaucoma screening were included, both in-comparison to in-person screening and analyzing teleglaucoma on its own. Outcome measures of teleglaucoma articles selected contained efficiency measures, specificity, sensitivity, and its ability to detect glaucoma, as well as patient benefits and cost data. Economic evaluations such as cost-effectiveness analysis and studies with costing data were also included.

The exclusion criteria was articles published prior to 1999 since teleglaucoma is fairly new and to be consistent with the teleglaucoma screening procedure, year 1999 was selected as a cut-off year. Additionally, non-research articles such as methodology papers, editorials, review articles, commentaries, and letters were excluded. Articles on diagnosis or prognosis, genetic screening, and teleophthalmology for ocular conditions other than glaucoma were eliminated.

**Figure 1:**
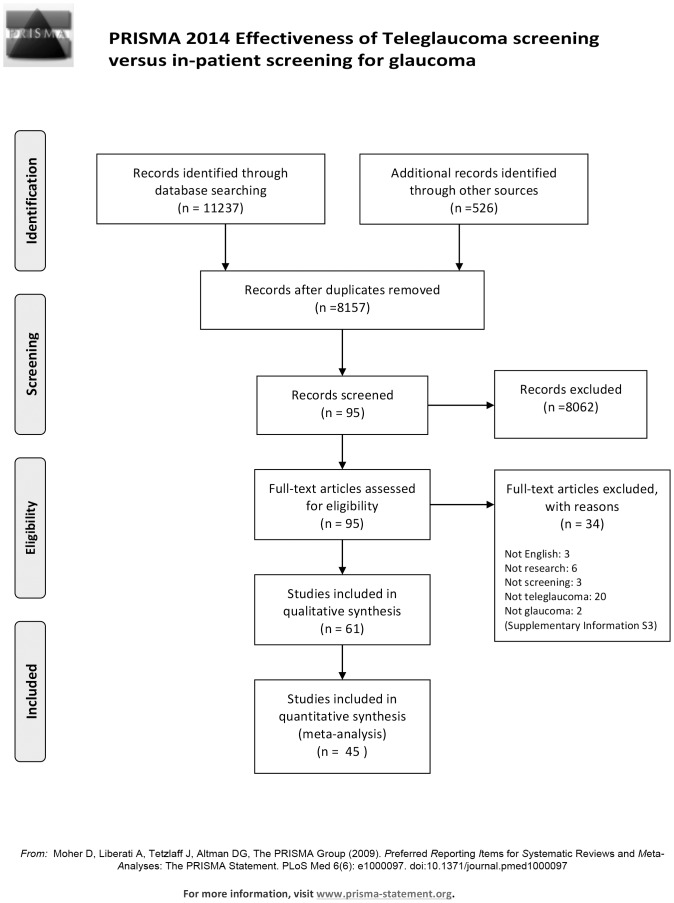
PRISMA diagram.

A total of 11237 articles were retrieved by searching various databases and an additional 526 were retrieved from hand searching and grey literature search which were then imported into EPPI 4.0 reference manager. Based on the inclusion and exclusion criteria, two reviewers independently reviewed all articles. After removing duplicate articles, 8157 articles were included for screening. Articles were screened by title, abstract, and full text in level 1, 2, and 3 screening respectively. After each level of screening, kappa statistics was calculated to measure reviewer's agreement. Additionally, if consensus was not reached by the two reviewers' then a third reviewer intervened to solve disagreements on article eligibility. The agreement between the two reviewers was excellent (kappa  = 0.86). The PRISMA diagram demonstrating the selection process is displayed in Figure 1 (Table S3).

### Quality Assessment Strategy

Articles were assessed using the Grading of Recommendations, Assessment, Development, and Evaluation (GRADE) guidelines for publication bias, risk of bias, imprecision, inconsistency, and indirectness [15]–[20]. Articles were graded as either low, moderate, or high quality of evidence. The results indicated that 17 articles were high quality, 13 were moderate quality, and 15 articles were graded as low quality of evidence. Despite the quality of evidence, all articles were included in the analysis.

### Data Extraction Strategy

Qualitative and quantitative data necessary for analysis was obtained from each article. Information on study location, design, effect measures (sensitivity and specificity), percentage of glaucoma diagnosed, service times, image quality, visual acuities, ophthalmic characteristics, and costs were collected. One reviewer extracted data using an excel template. Authors were emailed to obtain missing relevant information. All databases were updated with new information from respective authors. Additional current costing data was provided by ophthalmic equipment vendors INNOVA, Topcon, and Ocular Health Network. Costs were converted to 2014 US dollars [21]. This research study has no financial relationships, investments, or sponsorship related to the cited commercial vendors.

### Data Analysis

Data was synthesized and analyzed using STATA 13. When studies reported estimates as range or p-value or multiple estimates, mean and standard deviation (SD) were derived. Hierarchical logistic regression was used to determine the pooled estimates of sensitivity and specificity of teleglaucoma and in-person examination. A graphical representation of the summary estimates was presented in a Hierarchical Summary Receiver Operating Characteristic (HSROC) curve with 95% confidence intervals and 95% prediction regions.

The positive/negative likelihood ratios (LR+/LR−) were calculated using bivariate models to generate estimates of the likelihood of a positive/negative test in a glaucoma/non-glaucoma patient. From this result the diagnostic odds ratio (DOR) was calculated to determine the relative diagnostic effectiveness of teleglaucoma. DOR is the ratio of the odds of a positive screen test in a glaucoma case relative to the odds of a negative screen test in a non-glaucoma case [22].

Due to the variability of study effectiveness measures, not one article had a complete set of data. Missing data was treated as statistically missing values and not included in the analysis. Only articles with complete data were included in each analysis. Publication bias was assessed using funnel plots.

## Results

A total of 45 studies were included in this meta-analysis. Table 2 and Table 3 display the baseline characteristics of each study. Studies were conducted in fourteen different countries with representation in each continent. All articles were published between 1999 and 2014. The cumulated individuals of all studies were 101,512 participants. All studies were observational studies, as there were no randomized controlled trials conducted. Three studies contained economic evaluations or cost-effectiveness analysis. Of the 45 studies, 16 compared teleglaucoma to in-person examination. The other 29 studies analyzed teleglaucoma without comparison or was an evaluation of different teleglaucoma equipment. There was minimal variation in study populations; they included either glaucoma patients or patients who were at risk of glaucoma (based on diabetes status, family history of glaucoma, age, or ethnicity). Table 4 displays additional study details on demographics and study methods (glaucoma definition, pupil dilation, and number of field tests examined). Although there was some variation, less than 10% of studies reported these details. The main outcome measures were specificity and sensitivity (Table 5). Other included outcome measures (percentage of glaucoma diagnosed, referral rate, and proportion of images with poor quality) are displayed in table 5.

**Table 2:** Baseline characteristics of included studies – demographics.

Author (Year)	Location	Study Design	Sample Size	Population
Tuulonen et al. (1999) [11]	Finland	PC	70	Glaucoma patients
Eikelboom et al. (1999) [31]	Australia	PC	27	Glaucoma patients
Li et al. (1999) [35]	USA	PC	32	Diabetic adults
Yogesan et al. (1999) [36]	Australia	PC	27	Glaucoma clinic patients/suspected of glaucoma
Michelson et al. (2000) [37]	Germany	PC	10	Glaucoma-diagnosed patients
Yogesan et al. (2000) [38]	Indonesia	PC	14	Ophthalmic Clinic patients
Yogesan et al. (2000) [39]	Australia	PC	43	Ophthalmic Clinic patients
Gonzalez et al. (2001) [40]	Spain	PC	139	Ophthalmic Clinic patients
Sebastian et al. (2001) [41]	Spain	CS	74	Glaucoma suspects
Wegner et al. (2003) [42]	Germany	PC	1733	Not stated
Labiris et al. (2003) [43]	Greece	PC	1205	Glaucoma-diagnosed patients
Fansi et al. (2003) [44]	Canada	PC	33	Glaucoma suspects or diagnosed
Jin et al. (2003) [45]	Canada	CEA	339	Diabetic aboriginals
Chen et al. (2004) [46]	Taiwan	PC	113	Residents of area aged >40 years
de Mul et al. (2004) [47]	Netherlands	PC	1729	Optometrist patients at-risk for glaucoma
Ianchulev et al. (2005) [32]	USA	PC	33	Glaucoma suspects or diagnosed
Paul et al. (2006) [48]	India	PC	348	Rural residents at risk for glaucoma
Kumar et al. (2006) [33]	Australia	PC	107	Patients of the Eye Clinic
Kumar et al. (2007) [49]	New Zealand	PC	201	General eye examination clinic Patients
Khouri et al. (2007) [50]	Not Stated	CS	30	Glaucoma-diagnosed patients
Pasquale et al. (2007) [51]	USA	PC	350	Diabetic
Khouri et al. (2008) [52]	USA	PC	28	Glaucoma-diagnosed patients
deBont et al. (2008) [53]	USA	PC	1729	Optometrist patients at-risk for glaucoma
Sogbesan et al. (2010) [54]	Canada	CEA/PC	–	Optometrist patients at-risk for glaucoma
Anton-Lopez et al. (2011) [55]	Spain	CS	1599	At-risk for glaucoma
Khurana et al. (2011) [56]	India	CS	91698	Ophthalmic Clinic patients
Staffieri et al. (2011) [57]	Tasmania	PC	133	High risk (First degree relatives of diagnosed POAG)
Swierk et al. (2011) [58]	Germany	EE	–	Ophthalmic Clinic patients
Amin et al. (2012) [59]	Canada	PC	72	Glaucoma suspects or early stages of OAG
Shahid et al. (2012) [6]	USA	CS	341	Urban soup kitchen/homeless
Kassam et al. (2012) [9]	Canada	PC	257	At-risk for glaucoma or early-stage glaucoma
Gupta et al. (2013) [14]	India	PC	247	Ophthalmic Clinic patients
Damji et al. (2013) [60]	Canada	PC	71	Ophthalmic Clinic patients
Kiage et al. (2013) [13]	rural Africa	PC	309	Diabetic adults
Verma et al. (2013) [61]	Canada	RC	247	Optometrist-referred glaucoma suspects or early OAG
Ahmed et al. (2013) [62]	USA	RC	643	Diabetic and hypertensive
Arora et al. (2014) [12]	Alberta	PC	71	Glaucoma clinic patients/suspected of glaucoma

Legend: CS  =  Cross-Sectional Study, PC  =  Prospective Cohort Study, CEA  =  Cost-effectiveness Analysis, RCS  =  Retrospective Cohort Study, EE  =  Economic Evaluation, –  =  Not Stated.

**Table 3:** Baseline characteristics of included studies – intervention.

Author (Year)	Teleglaucoma Equipment	Comparator
Tuulonen et al. (1999) [11]	Canon CR5-45NM non-mydriatic fundus camera, slit-lamp, Panasonic video camera, HF II perimeter	In-person examination
Eikelboom et al. (1999) [31]	Nidek Nm-100 Handheld fundus camera	Teleglaucoma only
Li et al. (1999) [35]	Non-mydriatic retinal camera. Digital images	Image Quality of Teleglaucoma
Yogesan et al. (1999) [36]	Portable fundus camera, Nidek NM100	Teleglaucoma only
Michelson et al. (2000) [37]	Self-tonometry portable device called Ocuton, PalPilot, IOP curve	Teleglaucoma only
Yogesan et al. (2000) [38]	Handheld fundus camera (NM100)	Teleglaucoma only
Yogesan et al. (2000) [39]	DIO digital indirect ophthalmoscope, handheld fundus camera Nidek NM100, stereo fundus camera (Nidek 3D-x)	Teleglaucoma only
Gonzalez et al. (2001) [40]	Non-mydriatic fundus camera (canon CR6-45M)	In-person examination
Sebastian et al. (2001) [41]	C-20-5 FDT, Humphrey-Zeiss, & Topcon optic nerve head photographs	Teleglaucoma only
Wegner et al. (2003) [42]	Goldman applanation tonometer and mobile HRT	Teleglaucoma only
Labiris et al. (2003) [43]	Slit lamp, Octapus perimeter visual field, fundus camera, Optotype, air tonometer	In-person examination
Fansi et al. (2003) [44]	–	Healthy vs Glaucoma eyes
Jin et al. (2003) [45]	Tonometry	In-person examination
Chen et al. (2004) [46]	Digital 35-degree colour fundus images, non-mydriatic digital fundus camera (CR6-45, Canon)	In-person examination
de Mul et al. (2004) [47]	Nerve fibre analyser, GDx	In-person examination
Ianchulev et al. (2005) [32]	Peristat: self-test	In-person examination
Paul et al. (2006) [48]	–	Teleglaucoma only
Kumar et al. (2006) [33]	I-care tonometry	Teleglaucoma only
Kumar et al. (2007) [49]	–	In-person examination
Khouri et al. (2007) [50]	Digital stereo fundus camera - Nidek 3-Dx	Image Quality of Teleglaucoma
Pasquale et al. (2007) [51]	Topcon TRC NW-5S non-mydriatic retinal camera (Paramus) interfaced to a standard color video camera (Sony 970-MD)	Teleglaucoma only
Khouri et al. (2008) [52]	Non-mydriatic 45-deg camera, Canon Japan. DICOM image format	Image Quality of Teleglaucoma
deBont et al. (2008) [53]	Nerve fibre analyser, GDx	Image Quality of Teleglaucoma
Sogbesan (2010) [54]	–	In-person examination
Anton-Lopez et al. (2011) [55]	HRT, nerve-fibre analyzer (GDX-VCC), I-Care (rebound tonometry)	In-person examination
Khurana et al. (2011) [56]	–	Teleglaucoma only
Staffieri et al. (2011) [57]	–	Teleglaucoma only
Swierk et al. (2011) [58]	–	In-person examination
Amin et al. (2012) [59]	Slit lamp, IOP, CCT, visual field, anterior and stereo posterior segment photos and OCT	In-person examination
Shahid et al. (2012) [6]	8.2 megapixel non-mydriatic retinal camera	Teleglaucoma only
Kassam et al. (2012) [9]	Remote service - slit lamp, fundus photographs,	In-person examination
Gupta et al. (2013) [14]	Fundus Camera (Portcam II)	In-person examination
Damji et al. (2013) [60]	–	In-person examination
Kiage et al. (2013) [13]	Topcon 777	In-person examination
Verma et al. (2013) [61]	–	In-person examination
Ahmed et al. (2013) [62]	Topcon TRC non-mydriatic retinal camera, Tonopen	Teleglaucoma only
Arora et al. (2014) [12]	–	In-person examination

**Table 4:** Additional Details on Baseline Characteristics of Included Studies.

Author (Year)	Study Population Ethnicity	Glaucoma definition	Dilated pupil	# Field tests
Eikelboom et al. (1999) [31]	–	–	Yes	–
Yogesan et al. (1999) [36]	–	–	Yes	–
Yogesan et al. (2000) [38]	–	–	Yes	–
Yogesan et al. (2000) [39]	–	–	Yes	–
Ianchulev et al. (2005) [32]	15% White, 9% African American, 76% Hispanic	–	No	–
Chen et al. (2004) [46]	100% Asian	“The diagnosis of glaucoma was made according to the anatomical findings from the patient's optic nerve disc, and functional visual field examination by frequency-doubling perimetry (FDP). Intraocular pressure (IOP) was also evaluated. An elevated IOP was defined as over 17 mmHg (1 mmH = 133 Pa). Severe glaucoma was defined as an optic cup: disc ratio over 0.7 with an FDP defect or elevated IOP. Mild glaucoma was defined as an optic cup: disc ratio between 0.7 and 0.5, or disc asymmetry of over 20%, with an FDP defect or elevated IOP.”	–	–
Kumar et al. (2006) [33]	96% Caucasian, 4% Asian	IOP of 21 mmHg was threshold for suspected glaucoma	–	–
Paul et al. (2006) [48]	100% Indian	–	–	–
Kumar et al. (2007) [49]	–	In accordance with glaucoma screening protocol of Lions Eye Institute: Vertical cup disc ratio (VCDR) >0.5, IOP >21 mmHg, abnormal visual field related to glaucoma, and or disk asymmetry >0.2.	Yes	–
Pasquale et al. (2007) [51]	16% African American (of glaucoma suspects) 14% African American (Of non-glaucoma suspects)	“VFs were considered glaucomatous if the pattern deviation plot showed a nasal step, nasal depression, arcuate defect, paracentral loss that respected the horizontal meridian, or temporal wedge defects based on previously published criteria… Patients were designated as “no glaucoma” if the CDR was “<0.6 in both eyes and CDR asymmetry was <0.1 in the absence of reliable glaucomatous VFs. Patients were designated as having “glaucoma-suspicious optic discs” if the CDR was ”>0.6 in either eye or CDR asymmetry was >0.1 with or without reliable glaucomatous VFs. Patients with more subtle optic nerve changes were labeled as having glaucoma-suspicious optic discs if VFs were available and reliable and showed change consistent with glaucomatous loss.”	–	Three
Staffieri et al. (2011) [57]	–	“Subjects were classified as having definite glaucoma on the basis of characteristic optic nerve head changes (cup: disc ratio [CDR] outside the 97.5 percentile for the normal population or rim width less than 0.1 CDR at the superior and inferior poles of the disc) and definite visual field defect consistent with glaucoma. Individuals with stereoscopic disc photos consistent with structural damage but in whom field testing was unreliable or unobtainable were classified as glaucoma suspect.”	Yes	–
Khurana et al. (2011) [56]	100% Indian	–	–	–
Anton-Lopez et al. (2011) [55]	–	“2/3 Criteria were considered suspects and referred for glaucoma consultation: (1) global Moorefield's Regression Analysis borderline or outside normal limits, (2) Nerve Fibre Index >30, and tonometry >21 mmHg.”	–	–
Shahid et al. (2012) [6]	78% African American, 10% Caucasian, 6.7% Hispanic, 4.8% Other	–	Yes	One
Kiage et al. (2013) [13]	100% African	Category 1 diagnosis (structural and functional evidence): 2 out of 3 of the following: VCDR ≥0.7, focal glaucoma disc changes, VCDR asymmetry (≥0.2). Category 2 diagnosis (structural evidence with unproved field loss): 2 out of 3 of the following: VCDR ≥0.8, focal glaucoma disc changes, VCDR asymmetry ≥0.3. Category 3 diagnosis (optic disc not clearly seen): 1 of the following visual acuity <3/60 and IOP> 21 mmHg or visual acuity <3/60 and evidence of glaucoma surgery or medical records confirming glaucoma morbidity. Glaucoma suspect: one of the following IOP ≥23 mmHg, 1/3 of the glaucomatous optic neuropathy listed in category 2, glaucoma visual field defect only.	Yes	Three
Gupta et al. (2013) [14]	100% Indian	Glaucoma diagnosis based on disc findings VCDR of ≥0.7 or focal neuroretinal rim defect.	Yes	–

**Table 5:** Study relevant outcome measures.

Author (Year)	Specificity (%)	Sensitivity (%)	Percentage Glaucoma diagnosed	Percentage Referral Rate	Percentage of Image of Poor Quality
Li et al. (1999) [35]	–	–	–	–	18.8
Yogesan et al. (1999) [36]	84.5	82.5	–	–	–
Eikelboom et al. (1999) [31]	71.5	67	–	–	–
Yogesan et al. (2000) [39]	87	100	–	–	–
Gonzalez et al.(2001) [40]	–	–	7.9	–	13
Sebastian et al. (2001) [41]	–	–	2.7	–	4
Wegner et al. (2003) [42]	–	–		–	9.4
de Mul et al. (2004) [47]	58	82	4.6	11	–
Ianchulev et al. (2005) [32]	95.5	81.5	–	–	–
Kumar et al. (2006) [33]	98.8	38.1	–	–	–
Kumar et al. (2007) [49]	93.6	91.1	–	–	–
Pasquale et al. (2007) [51]	96	59	–	–	–
deBont et al. (2008) [53]	–	–	–	11	11
Staffieri et al. (2011) [57]	–	–	5	–	–
Anton-Lopez et al. (2011) [55]	–	–	1.9	7.7	–
Khurana et al. (2011) [56]	–	–	1.06	12.5	–
Shahid et al. (2012) [6]	–	–	32		–
Ahmed et al. (2013) [62]	–	–	–	19.4	5
Gupta et al. (2013) [14]	81.82	72.1	–	–	–
Kiage et al. (2013) [13]	89.6	41.3	14	–	24
Verma et al. (2013) [61]	–	–	31	31	
Arora et al. (2014) [12]	–	–	44	–	–

Costing data was given by nine studies and the quality of analysis of costing is displayed in table 6. Teleglaucoma costs vary by the capacity of the service and the type and amount of equipment. The current vendor estimate shows that the total costs for standard glaucoma equipment range from 89,703.53 to 123,164.55 US dollars (Table 6) [23], [24]. Additionally, to transfer images and patient test results securely to ophthalmologists electronically a service exists costing $62.13 US/month [21], [25]. This service allows teleglaucoma technicians and ophthalmologists to login electronically to attach, send, view and assess retinal images and patient test results.

**Table 6:** Quality of analysis for costing.

Author (Year)	Object	Costs ($)	Currency
Tuulonen et al. (1999) [11]	Fixed Costs		
	Fundus camera (1 unit)	200	FIM
	ISDN installation (3 units)	6.5	FIM
	Server computer (2 units for 5 years)	50	FIM
	Software application (2 units for 5yrs)	50	FIM
	Video slit-lamp (1 unit)	40	FIM
	Write off 10 years (3%)	40.62	FIM
	Use of teleophthalmology equipment	24.372	FIM
	Video conference equipment	84	FIM
	Write-off 5 years	18.342	FIM
	Automated perimetry – Humphrey	132	FIM
	Write off 10 years (3%)	15.474	FIM
	Other fixed costs		
	Service and updating	5	FIM
	Line costs per month	3.672	FIM
	Premise	1.608	FIM
	Utilities	1.608	FIM
	Other costs	7.133	FIM
Yogesan et al. (2000) [38]	Satellite phone	30000	EUR
	Mobile phone	3250	EUR
Jin et al. (2003) [45]	Total expenditure capital	160260	CAN
	Operating costs per 1 year	348665	CAN
	Projected 2005 Costs	385226	CAN
	Operating costs amortized over 5 years	32052	CAN
	Operating costs amortized over 5 years per diabetic case	1231	CAN
	Professional and Lab Fees	291	CAN
	Costs per patient	1231	CAN
	Travel costs	805	CAN
	Escort travel expenses	340	CAN
Chen et al. (2004) [46]	Costs per detected case	10	US
Ianchulev et al. (2005) [32]	Costs per targeted glaucoma screening	60	US
	Costs per detected case	1000	US
Sogbesan (2010) [54]	Patient savings	2527	CAN
Anton-Lopez et al. (2011) [55]	Incremental Costs	24150	EUR
	Costs per detected case	1420	EUR
	Primary Care visit	15	EUR
	General Ophthalmic Visit	18	EUR
	Ophthalmic Visit with tests	52	EUR
	Glaucoma Consultation	26	EUR
Swierk et al. (2011) [58]	Medical Care	291.21	EUR
	Accommodation costs	280	EUR
	Costs per patient	288.72	EUR
Ahmed et al. (2013) [62]	Equipment costs (digital retinal camera, Tonopen and computer)	46000	US
Vendor Estimates (2014) [23], [24]	OCT	48,000–49,000	CAN
	Slit Lamp	7,420–19,990	CAN
			
	Tonometer		
	Slit lamp mounted	1,400–2,400	CAN
	Non-contact	8,995	CAN
			
	Retinal Camera	27,900–27, 995	CAN
	Visual Field Analyser	16,340–32,420	CAN
	**TOTAL RANGE:**	**89,703.53–123,164.55**	US
Ocular Health Network (2014) [25]	Imaging Transfer Service	70/Month	CAN

Costing data from the literature shows the cost per detected case of glaucoma ranged from $13.03–2020.96 US after conversion to US dollars and adjusted for inflation to 2014 costs (Table 7) [26]. The mean cost is $1098.67 US for every case of glaucoma detected (n = 3) (Table 7). The mean cost of teleglaucoma per patient screened was $922.77 US (n = 2) (Table 7).

**Table 7:** Teleglaucoma estimated 2014 unit costs.

Author (Year)	Cost per detected case ($US) (Adjusted for inflation to 2014 costs)	Inflation Rate (%)	Cost per patient ($US) (Adjusted for inflation to 2014 costs)	Inflation Rate (%)
Jin et al. (2003) [45]	–	–	1434.63	25.49
Chen et al. (2004) [46]	13.03	30.32	–	–
Ianchulev et al. (2005) [32]	1262.02	26.2	–	–
Anton-Lopez et al. (2011) [55]	2020.96	5.89	–	–
Swierk et al. (2011) [58]	–	–	410.91	5.89
**Mean costs**	**1098.67**		**922.77**	

Another necessary costing aspect is the ophthalmologist fee for glaucoma consultation. The ophthalmologist may be compensated for each teleglaucoma referral or time spent on teleophthalmology consultations. Compensation varies by states and/or provinces, government legislation, and available private grants. In the United States, *Medicare* and *Medicaid* provide several reimbursement programs for physicians delivering telemedicine consultations [27], [28]. In Ontario, Canada, the compensation for the fee-for service model, is $16.00 CAN per ophthalmic referral [29]. The physician liable for teleglaucoma consultations must be a licensed ophthalmologist in both the area of the service and the patient. Physicians must hold liability coverage appropriate to state/provincial laws. In Canada, the *Canadian Medical Protective Association* provides ophthalmologists with liability coverage for teleophthalmology [30].

**Figure 2:**
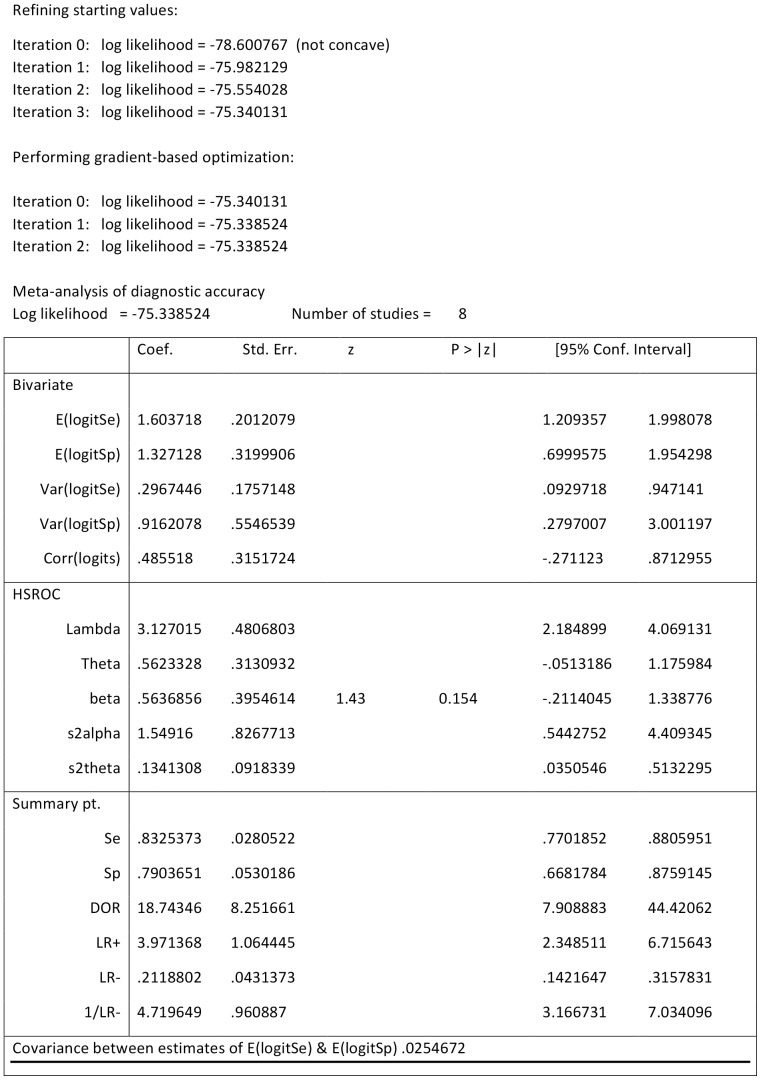
Hierarchical logistic regression results.

**Figure 3:**
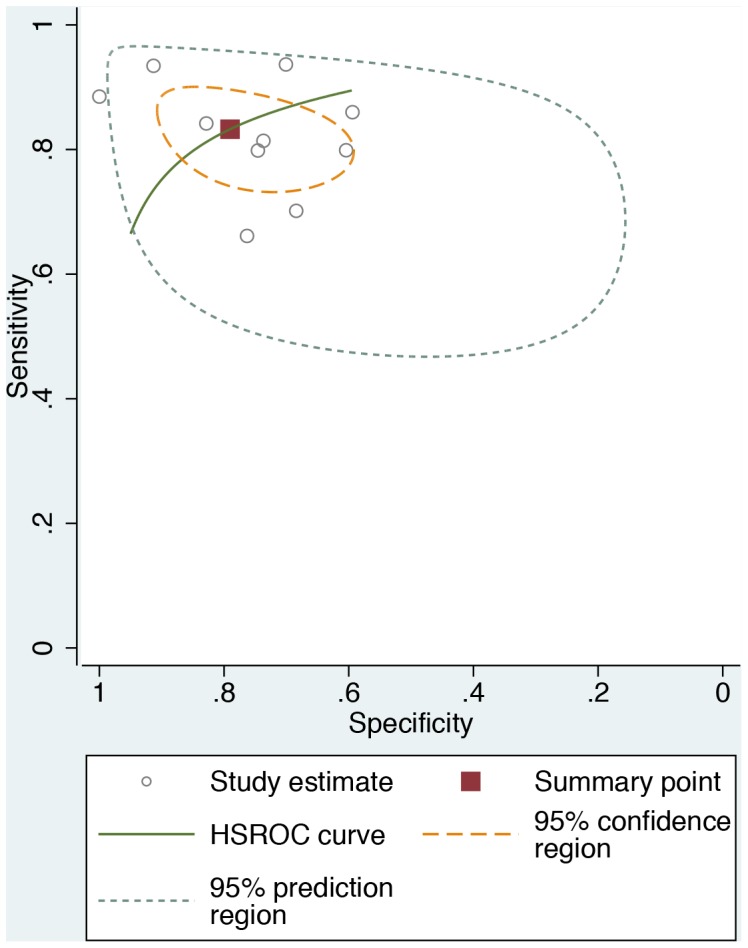
Hierarchical Summary Receiver Operating Characteristic (HSROC) plot.

Ten studies had complete data necessary to conduct the analysis for teleglaucoma diagnostic accuracy. The summary estimate for sensitivity was 0.833 [95% CI 0.77, 0.88] and specificity was 0.79 [95% CI 0.668, 0.875] for glaucoma screening using optic nerve examinations (Figure 2). The summary estimates indicate that teleglaucoma correctly detects 83.3% of glaucoma cases and correctly classifies 79% of those without glaucoma as glaucoma-negative. Figure 3 displays each study estimate and the summary estimate with its associated confidence intervals and the generated HSROC curve. The distribution of the studies in the plot demonstrates the variability of both specificity and sensitivity amongst studies. Six studies fall outside of the 95% confidence interval of the summary estimate. The 95% prediction region is the estimate of future observations. The results demonstrate a fairly wide prediction region for both true predictions of specificity and sensitivity, with greater variability expected for specificity.

The study populations used to assess diagnostic accuracy were those at-risk of glaucoma (based on diabetes status, family history, age, ethnicity, etc.), optometrist and ophthalmic clinic patients, and patients who were glaucoma suspects (Table 1). One study reported its study population as glaucoma patients only (Table 1) and contrary, this study had one of the lower reported scores for diagnostic accuracy: specificity was 71.5% and sensitivity was 67% (Table 5) [31].

The diagnostic tools of the included studies varied slightly (Table 8). Eight out of the ten studies analyzed for sensitivity and specificity used at minimum optic nerve examinations as part of the screening process (Table 8). The other two studies reported using IOP or visual field defects as the methods to detect glaucoma suspects (Table 8). For these studies which did not include fundus photographs, the sensitivity and specificity were 81.5% and 95.5% respectively for glaucoma screening using only visual field and 38.1% and 98.8% respectively for glaucoma screening using IOP and Orbscan Topography (Table 5) [32], [33].

**Table 8:** Study ophthalmic examinations.

Author (Year)	Examination tests
Li et al. (1999) [35]	Optic disc photographs, VCDR
Yogesan et al. (1999) [36]	VCDR
Eikelboom et al. (1999) [31]	VCDR
Yogesan et al. (2000) [39]	Fundus images, H/VCDR, radial rim measurements
Gonzalez et al.(2001) [40]	Fundus images
Sebastian et al. (2001) [41]	Visual acuity, IOP, FDT, optic nerve head photographs
Wegner et al. (2003) [42]	HRT, IOP, OCT
de Mul et al. (2004) [47]	IOP, nerve fibre indicators
Ianchulev et al. (2005) [32]	HVF, visual acuity
Kumar et al. (2006) [33]	IOP, CCT, ACT
Kumar et al. (2007) [49]	IOP, FDT, VCDR, disc asymmetry, visual field, fundus photographs
Pasquale et al. (2007) [51]	IOP, CDR, Humphrey visual field, comprehensive eye examination
deBont et al. (2008) [53]	Nerve fiber indicators, fundus photographs, IOP
Staffieri et al. (2011) [57]	Visual acuity, refractive status, visual field testing, IOP, CCT, stereoscopic optic disc photographs
Anton-Lopez et al. (2011) [55]	IOP, HRT, nerve fibre indicators
Khurana et al. (2011) [56]	–
Shahid et al. (2012) [6]	IOP, optic nerve head appearance and asymmetry, nerve fibre layer dropouts
Ahmed et al. (2013) [62]	Fundus images, CDR, IOP
Gupta et al. (2013) [14]	Fundus photographs
Kiage et al. (2013) [13]	Slit lamp examination, focal glaucoma damage, VCDR, IOP, FDT, fundus images, visual fields
Verma et al. (2013) [61]	Stereoscopic optic nerve images, visual fields, ancillary tests, IOP, OCT, and HRT
Arora et al. (2014) [12]	OCT, HRT, stereo-nerve photographs, FDT, HVF, OCT, IOP

**Legend:** VCDR  =  vertical cup-to-disc ratio, HCDR  =  horizontal cup-to-disc ratio, IOP  =  intraocular pressure, FDT  =  frequency doubling technology, CCT  =  central corneal thickness, HRT  =  Heidelberg Retinal Tomography, CDR  = cup-to-disc ratio, HVF  =  Humphrey Visual Field, ACT  =  anterior chamber depth, POAG  =  primary open angle glaucoma, OAG  =  open angle glaucoma.

Three studies reported sensitivity and specificity of in-person examination. The weighted mean of sensitivity was 74.9±27.6% (n = 3) and specificity was 88.8±10.3% (n = 3) for in-person examination. The summary estimates indicate that in-person examination correctly detects 74.9% of glaucoma cases and correctly classifies 88.8% of those without glaucoma as glaucoma-negative.

The positive likelihood ratio was 3.97 [95% CI: 2.3–6.7] while the negative likelihood ratio was 0.21 [95% CI: 0.14–0.32] (Figure 2). This demonstrates that the likelihood of a positive screen test in a glaucoma case is greater than the likelihood of a negative screen test in a non-glaucoma case. In addition, the positive likelihood ratio is greater than one and thus the positive screen test is associated with glaucoma. Since the negative likelihood ratio is less than one, the negative screen test is associated with the absence of disease [22]. The effectiveness of the diagnostic accuracy of teleglaucoma was given by the DOR, which was 18.7 [95% CI: 7.9–44.4] (Figure 2). The relative odds of a positive screen test in glaucoma cases are 18.7 times more likely than a negative screen test in a non-glaucoma case. Since the DOR was greater than one the test is discriminating between true positives and true negatives correctly [22].

There was insufficient data to conduct hierarchical logistic regression on the percentage of glaucoma diagnosed. Three of the 45 studies reported percentage of glaucoma diagnosed in both teleglaucoma and in-person examination necessary for analysis. The mean percentage of glaucoma diagnosed was 13.4% for teleglaucoma and 7.8% for in-person examination which suggests that teleglaucoma is capable of detecting more cases of glaucoma.

Other effectiveness measures of teleglaucoma were analyzed such as variables of healthcare service quality. The mean percentage of patients referred to specialist for consultation was 12.5±7.8% (n = 6). The mean percentage of images that were of poor quality was 10.4±6.7% (n = 7). It took a mean time of 75.6±87.7 seconds (n = 4) to process the teleglaucoma images. Timing associated with teleglaucoma service is another measure of quality. The mean time for screening was 8.8±5.1 minutes (n = 3). The time reported for ophthalmologist to make diagnosis was 34 minutes (n = 1). The mean reporting time was 7.6±2.6 minutes (n = 6). Teleglaucoma gave a reduction for patient travel time of 61.23 hours (n = 1). Teleglaucoma had a mean access time (time from patient being referred to the date visit is booked) of 59.7±9.9 minutes (n = 4) in comparison to 73.7±29.8 minutes (n = 4) for in-person examination. The mean cycle time (time from registration until patient leaves clinic) for teleglaucoma was 81.7±6 minutes (n = 2), which was less than that of in-person examination, 116±2.5 minutes (n = 2). The mean proportion of patient satisfaction with teleglaucoma was 47.3±8.8% (n = 2) while only 42% (n = 1) were satisfied with in-person examination.

## Discussion

Telemedicine has demonstrated good use for offering glaucoma services to people of remote areas. Teleglaucoma is beneficial to remote areas as the physician is not required to see patients in person, which reduces wait times and shortens the length of ophthalmic consultations. Teleglaucoma avoids long distance travel and time wasted on commute. The results of the pooled estimates for diagnostic accuracy have shown teleglaucoma to be more sensitive and less specific than in-person examinations. Teleglaucoma is advantageous at detecting true positive cases of glaucoma, but has a higher rate of false positives in comparison to in-person examination. With very high DOR estimates, it is suggested that teleglaucoma can accurately discriminate screen tests. Teleglaucoma has demonstrated capability to detect glaucoma cases that may not have been detected during in-person examination. Glaucoma progresses without patient awareness and it is usually detected at the advanced stages. Thus teleglaucoma serves as a tool for early detection of glaucoma. If caught earlier and with treatment, glaucoma can be effectively managed and can result in the preservation of vision.

Telemedicine for glaucoma can have several combinations of examinations and measurements used for glaucoma screening. Examination of fundus photographs are commonly used for teleglaucoma screening. Four of the ten studies analyzed used only fundus examinations while another four studies included IOP, CCT, visual field loss, and visual acuity, in addition to fundus photograph examinations (Table 8). Two studies did not use fundus photograph examination but rather visual acuity, IOP, CCT, and ACT (Table 8). However, this is based on studies who explicitly stated the terms for ophthalmic examination. Some studies reported “comprehensive eye examinations” were performed, but did not explicitly state which examinations were performed, thus assumptions cannot be made. The use of different tests for glaucoma screening can potentially bias the results as the more diagnostic tools used during screening results in a greater probability of correct diagnosis naturally. However, the results did not show any significant differences in accuracy with studies which reported using multiple diagnostic tools. Interestingly, the specificity and sensitivity values reported ranged independent of the number and the type of examination used for teleglaucoma (Table 4 and Table 8).

The combinations of examinations are dependent on financial and resource limitations of the hosting organization and can vary from small programs to very large programs. It is dependent on the target goals and target populations of the organization. However, the standard examinations recommended for glaucoma screening are those that can evaluate visual field defects, IOP, and the biological structure and function of the optic nerve. These include HRT, OCT, optic disc photography, RNFL photography, as well as FDT, tonometry, and perimetry [34]. There were limitations within the study. Insufficient data reported was a major limitation of the meta-analysis, although authors were contacted for additional information. Nevertheless, the key goal was to systematically review the literature on tele-glaucoma and in-person screening and perform the meta-analysis. With small samples sizes there was not enough power to show statistical or clinical significance. Different comparators were reported by studies and to ensure internal validity, only studies with exact comparators were analyzed together. This was one of the reasons for reduced sample sizes for the analysis. However, our analysis does provide information on diagnostic accuracy of teleglaucoma, its capability to detect glaucoma, and to detect negative and positive cases correctly. It demonstrates teleglaucoma has the potential as a screening device to detect a greater amount of cases than in-person examination. Since teleglaucoma is an active screening, it suggests glaucoma cases are detected at earlier stages. However the significance of this difference is limited by the number of comparative studies. The majority of the studies were non-comparative which, in addition, limits the significance of the relative effectiveness to in-person examination.

Teleglaucoma has been evaluated in many different ways: diagnostic accuracy, cost reduction, technological capabilities (image quality, image transmission speed, etc.), reduction of patient and health care provider time, and convenience. Thus many studies focus on only part of the effectiveness. As a result, there is insufficient data when summarizing all of the studies together. This has proven the need for more research literature on the diagnostic accuracy of teleglaucoma and its ability to detect glaucoma in comparison to in-person examination. There is a need for research on the follow-up of detected cases and long-term effects of teleglaucoma. In addition, better quality of evidence through randomized controlled trials is recommended. There are implications for cost-effectiveness analyses. Although, costing data suggests cost savings for patients' time and travel with teleglaucoma, a thorough costing of current health care expenditure is required to determine its overall cost-effectiveness from the scope of the healthcare system.

Teleglaucoma is beneficial to offering services in underserviced regions and rural areas. It considerably reduces patient access times and cycle times. The time required for service is shorter than in-person examination and physician commitments are reduced. As a result teleglaucoma saves costs to patients and costs to the health care system as a whole.

## Supporting Information

Table S1 **Systematic review search strategy.**
(DOCX)

Table S2 **PRISMA checklist.**
(DOCX)

Table S3 **Excluded full-text articles.**
(DOCX)
