# Correction: The Effectiveness of Teleglaucoma versus In-Patient Examination for Glaucoma Screening: A Systematic Review and Meta-Analysis

**DOI:** 10.1371/journal.pone.0118688

**Published:** 2015-03-05

**Authors:** The PLOS ONE Staff

There is an error in the second author’s name. The correct name is: Maya M. Jeyaraman. The correct citation is: Thomas S-M, Jeyaraman MM, Hodge WG, Hutnik C, Costella J, Malvankar-Mehta MS. (2014) The Effectiveness of Teleglaucoma versus In-Patient Examination for Glaucoma Screening: A Systematic Review and Meta-Analysis. PLoS ONE 9(12): e113779. doi:10.1371/journal.pone.0113779
