# Supplementary material for: The Effectiveness of Teleglaucoma versus In-Patient Examination for Glaucoma Screening: A Systematic Review and Meta-Analysis
Source: PLoS One. 2014 Dec 5;9(12):e113779. doi: 10.1371/journal.pone.0113779 (PMC4257598; doi:10.1371/journal.pone.0113779)
Supplement: Table S1 — Systematic review search strategy. (DOCX) [file pone.0113779.s001.docx]

**S1 Table 1: Systematic Review Search Strategy**

| **DATABASE** |  | | **SEARCH TERMS** |
| --- | --- | --- | --- |
| OVID Medline |  | |  |
|  | 1 | | Exp Glaucoma/ OR Intraocular Pressure/ OR Ocular hypertension/ |
|  | 2 | | Glaucoma* OR Intraocular pressure OR Intra-ocular pressure OR Intraocular hypertension OR Intra-ocular hypertension OR Intra-ocular tension OR Intraocular tension OR Ocular hypertension OR Ocular tension OR Eye tension OR Eye pressure |
|  | 3 | | 1 OR 2 |
|  | 4 | | Remote consultation/ OR Telemedicine/ OR Telepathology/ OR Mobile Health Units/ OR Community Pharmacy Services/ |
|  | 5 | | Automated detection OR Teleglaucoma OR Telescreen* OR Teleophthalm* OR Tele-ophthalm* OR Tele-glaucoma OR Telemedicine OR Tele-medicine OR digital indirect ophthalmoscop* OR Telemonitor* OR Tele-monitor* OR Teleconsult* OR Tele-consult* OR Telediagnos* OR Tele-diagnos* OR Telehealth OR Tele-health OR Mobile health OR eHealth OR Automated Perimetry Exam* |
|  | 6 | | 4 OR 5 |
|  | 7 | | Diagnosis/ OR Early diagnosis/ OR Diagnostic Techniques, Ophthalmological/ OR Tonometry, Ocular/ OR Diagnosis.fs. OR Vision screening/ OR Mass screening/ OR Visual Field Tests/ |
|  | 8 | | Diagnos* OR Screen* OR Tonomet* OR Detect* OR Perimetr* OR Campimetr* OR Visual field test* OR Oculplethysmograph* OR Vision test* OR Early diagnosis |
|  | 9 | | 7 OR 8 |
|  | 10 | | 3 AND 6 AND 9 |
|  | RESULTS | | 86 |
|  |  | | Ovid MEDLINE(R) In-Process & Other Non-Indexed Citations, Ovid MEDLINE(R) Daily and Ovid MEDLINE(R) 1946 to Present |
| OVID EBMASE |  | | |
|  | 1 | Exp glaucoma/ OR Intraocular pressure abnormality/ OR Intraocular pressure/ | |
|  | 2 | Glaucoma* OR Intraocular pressure OR Intra-ocular pressure OR Intraocular hypertension OR Intra-ocular hypertension OR Intra-ocular tension OR Intraocular tension OR Ocular hypertension OR Ocular tension OR Eye tension OR Eye pressure | |
|  | 3 | 1 OR 2 | |
|  | 4 | Telemedicine/ OR Telehealth/ OR Telediagnosis/ OR Telepathology/ OR Teleconsultation/ OR Telemonitoring/ OR computer assisted perimetry/ | |
|  | 5 | Automated detection OR Teleglaucoma OR Telescreen* OR Teleophthalm* OR Tele-ophthalm* OR Tele-glaucoma OR Telemedicine OR Tele-medicine OR digital indirect ophthalmoscop* OR Telemonitor* OR Tele-monitor* OR Teleconsult* OR Tele-consult* OR Telediagnos* OR Tele-diagnos* OR Telehealth OR Tele-health OR Mobile health OR eHealth OR Automated Perimetry Exam* | |
|  | 6 | 4 OR 5 | |
|  | 7 | diagnosis/ OR early diagnosis/ OR diagnostic accuracy/ OR diagnostic test accuracy study/ OR diagnostic value/ OR perimetry/ OR oculoplethysmography/ OR vision test/ | |
|  | 8 | Diagnos* OR Screen* OR Tonomet* OR Detect* OR Perimetr* OR Campimetr* OR Visual field test* OR Oculplethysmograph* OR Vision test* OR Early diagnosis | |
|  | 9 | 7 OR 8 | |
|  | 10 | 3 AND 6 AND 9 | |
|  |  | Embase Classic+Embase 1947 to 2014 March 11 | |
| **CINAHL** |  | | |
|  | 1 | (MH "Glaucoma+") OR (MH "Ocular Hypertension") OR (MH "Intraocular Pressure") | |
|  | 2 | Glaucoma* OR Intraocular pressure OR Intra-ocular pressure OR Intraocular hypertension OR Intra-ocular hypertension OR Intra-ocular tension OR Intraocular tension OR Ocular hypertension OR Ocular tension OR Eye tension OR Eye pressure | |
|  | 3 | 1 OR 2 | |
|  | 4 | (MH "Telehealth") OR (MH "Telemedicine") OR (MH "Remote Consultation") OR (MH "Telepathology") OR (MH "Mobile Health Units") | |
|  | 5 | Automated detection OR Teleglaucoma OR Telescreen* OR Teleophthalm* OR Tele-ophthalm* OR Tele-glaucoma OR Telemedicine OR Tele-medicine OR digital indirect ophthalmoscop* OR Telemonitor* OR Tele-monitor* OR Teleconsult* OR Tele-consult* OR Telediagnos* OR Tele-diagnos* OR Telehealth OR Tele-health OR Mobile health OR eHealth OR Automated Perimetry Exam* | |
|  | 6 | 4 OR 5 | |
|  | 7 | (MH "Diagnosis") OR (MH "Diagnostic Services") OR (MH "Diagnosis, Eye") OR (MH "Tonometry") OR (MH "Vision Screening") OR (MH "Vision Tests") OR (MH "Perimetry") OR (MH "Early Diagnosis") | |
|  | 8 | Diagnos* OR Screen* OR Tonomet* OR Detect* OR Perimetr* OR Campimetr* OR Visual field test* OR Oculplethysmograph* OR Vision test* OR Early diagnosis | |
|  | 9 | 7 OR 8 | |
|  | 10 | 3 AND 6 AND 9 | |
| Cochrane, Web of Science, BIOSIS, Dissertations and Thesis, Canadian Health Research Collection |  | | |
|  | 1 | Glaucoma* OR Intraocular pressure OR Intra-ocular pressure OR Intraocular hypertension OR Intra-ocular hypertension OR Intra-ocular tension OR Intraocular tension OR Ocular hypertension OR Ocular tension OR Eye tension OR Eye pressure | |
|  | 2 | Automated detection OR Teleglaucoma OR Telescreen* OR Teleophthalm* OR Tele-ophthalm* OR Tele-glaucoma OR Telemedicine OR Tele-medicine OR digital indirect ophthalmoscop* OR Telemonitor* OR Tele-monitor* OR Teleconsult* OR Tele-consult* OR Telediagnos* OR Tele-diagnos* OR Telehealth OR Tele-health OR Mobile health OR  eHealth OR Automated Perimetry Exam* | |
|  | 3 | Diagnos* OR Screen* OR Tonomet* OR Detect* OR Perimetr* OR Campimetr* OR Visual field test* OR Oculplethysmograph* OR Vision test* OR Early diagnosis | |
|  | 4 | 1 AND 2 AND 3 | |
