# Supplementary material for: The Effectiveness of Teleglaucoma versus In-Patient Examination for Glaucoma Screening: A Systematic Review and Meta-Analysis
Source: PLoS One. 2014 Dec 5;9(12):e113779. doi: 10.1371/journal.pone.0113779 (PMC4257598; doi:10.1371/journal.pone.0113779)
Supplement: Table S3 — Excluded full-text articles. (DOCX) [file pone.0113779.s003.docx]

**Supplementary Information S3 – PRISMA Diagram**

1. Adler W, Warntges S, Lausen B, Michelson G (2010) Prevalence of glaucomatous optic nerve atrophy among a working population in Germany diagnosed by a telemedical approach. Telemedizinische Erfassung der Pravalenz der glaukomatosen Optikusatrophie in einer arbeitenden Bevolkerungsgruppe 227(11): 905-11.
2. Goldberg, Walt, Lee (2004) Comparing treatment costs associated with screening for glaucoma vs not screening: Evaluating the economic impact of the new HEDIS measure. VALUE IN HEALTH 7(6SU S).
3. Goldberg, Walt (2006) Cost-effectiveness of a glaucoma-screening program: A model evaluating the relative clinical and economic impact in commercial vs. senior member populations. VALUE IN HEALTH 9(3).
4. Hall, Hennessy, Barton, Coroneo (2005) Teleophthalmology-assisted corneal foreign body removal in a rural hospital. TELEMEDICINE JOURNAL AND E-HEALTH 11(1).
5. Lammer, Groh, Michelson (2004) Telematic-assisted self-tonometry (TAS). Telematisch assistierte Selbsttonometrie (TAS). Der Ophthalmologe : Zeitschrift der Deutschen Ophthalmologischen Gesellschaft 101(8): 813-8.
6. Lehmann, Mardin, Martus, Bergua (2008) 3D vs 2D qualitative and semi-quantitative evaluation of the glaucomatous optic disc atrophy using computer-assisted stereophotography. EYE 22(5).
7. Mah Sharon (2011) A Case Study of Telehealth Usage in Three First Nation Communities: Understanding the Role of Technology Users in Health Care Practice. University of Calgary (Canada).
8. Rendell, Burns, Murdoch (2000) Patients' satisfaction with teleconsultation. Ophthalmic Nursing: International Journal of Ophthalmic Nursing 4(2): 12-15.
9. Schargus, Michelson, Grehn (2011) Electronic patient records and teleophthalmology. Part 2: concrete projects in ophthalmology. Elektronische Patientenakte und Teleophthalmologie. Teil 2: Konkrete ophthalmologische Projekte. Der Ophthalmologe : Zeitschrift der Deutschen Ophthalmologischen Gesellschaft 108(7): 687-696.
10. U.S. Preventive Services Task Force (2005) Screening for glaucoma: recommendation statement. American Journal for Nurse Practitioners 9(9): 49.
11. Sharma A Corona, E Mitra, S Nutter, BS; (2006) Early detection of glaucoma using fully automated disparity analysis of optic nerve head (ONH) from stereo fundus images - art. no. 61440H. Medical Imaging 2006: Image Processing, Pts 1-3SE PROCEEDINGS OF THE SOCIETY OF PHOTO-OPTICAL INSTRUMENTATION ENGINEERS (SPIE). 6144.
12. Sim, Goh (1999) Screening for glaucoma in the Chinese elderly population in Singapore. Singapore medical journal 40(10): 644-7.
13. Skorkovska, Kelbsch, Blumenstock, Wilhelm, Wilhelm (2012) Glaucoma Screening by Means of Pupil Campimetry. KLINISCHE MONATSBLATTER FUR AUGENHEILKUNDE 229(11).
14. Stephen, Benjamin (2013) The East London glaucoma prediction score: web-based validation of glaucoma risk screening tool. INTERNATIONAL JOURNAL OF OPHTHALMOLOGY 6(1).
15. Stoutenbeek, Jansonius (2006) Glaucoma screening during regular optician visits: can the population at risk of developing glaucoma be reached?. British Journal of Ophthalmology 90(10): 1242-1244.
16. Streiner DL, Norman GR (2010) Mass screening: when does it make sense?. Community Oncology 7(2): 93-95.
17. (2013) Summaries for patients. Screening for Glaucoma: U.S. Preventive Services Task Force recommendation statement. Annals of Internal Medicine 159(7): I-28.
18. Syed, Radcliffe, De Moraes, Liebmann, Ritch (2011) Detection of progressive glaucomatous optic neuropathy using automated alternation flicker with stereophotography. Archives of ophthalmology 129(4): 521-2.
19. Szirth, Shahid, Khouri, Patel, Horan, Giliberti, Kaiser, Bhagat (2005) Tele-screening can help identify vision-threatening diseases. Ocular Surgery News 23(18): 86.
20. Tatemichi, Nakano, Tanaka, Hayashi, Nawa, Miyamoto, Hiro, Iwasaki, Sugita, Glaucoma Screening Project (GSP) Study Group (2002) Performance of glaucoma mass screening with only a visual field test using frequency-doubling technology perimetry. Erratum appears in Am J Ophthalmol. 2003 Sep;136(3):592]. 134(4): 529-37.
21. Tatemichi, Nakano, Tanaka, Hayashi, Nawa, Iwa (2003) Laterality of the performance of glaucoma mass screening using frequency-doubling technology. JOURNAL OF GLAUCOMA. 12(3): 221-5.
22. Tchabi, Doutetien, Amoussouga, Babagbeto, Lawani, Deguenon, Bassabi (2005) Intraocular pressure in the Benin: screening for primary open-angle glaucoma Le tonus oculaire chez les Beninois: depistage du glaucome primitif a angle ouvert. Journal francais d'ophtalmologie 28(6): 623-6.
23. Tennant, Rudnisky, Greve, Hinz, Smith (2004) The Prevalence of Diabetic Retinopathy and other Eye Diseases in Inuit Peoples of the Northwest Territories: A Teleophthalmology Pilot Project. ARVO Meeting Abstracts 45(5): 4132.
24. Thapa, Kelley, Rens, Paudyal, Chang (2008) A novel approach to glaucoma screening and education in Nepal. BMC ophthalmology 8: 21.
25. Thomas, Parikh, Paul, Muliyil (2002) Population-based screening versus case detection. Indian journal of ophthalmology 50(3): 233-7.
26. Thomas, Parikh, Muliyil, Bhat, George (2004) Validation of test duration as a screening criterion for frequency doubling perimetry. American journal of ophthalmology 137(3): 562-3.
27. Tong, Azen, Varma (2002) Screening for glaucoma and other ocular disease using SITA standard and full threshold Humphrey visual field tests: The Los Angeles Latino Eye Study (LALES). INVESTIGATIVE OPHTHALMOLOGY & VISUAL SCIENCE Annual Meeting of the Association for Research in Vision and Ophthalmology MAY 05-10, 2002 FT LAUDERDALE FLORIDA Assoc Res Vis & Ophthalmol. 43SU 1MA 2177.
28. Toth, Kothy, Hollo (2008) Accuracy of scanning laser polarimetry, scanning laser tomography, and their combination in a glaucoma screening trial. Journal of glaucoma 17(8): 639-46.
29. Turner, Graham, (2013) Potential effects of systematic errors in intraocular pressure measurements on screening for ocular hypertension. Eye (Basingstoke) 27(4): 502-506.
30. US Preventive Services Task Force (2005) Screening for glaucoma: recommendation statement. Annals of family medicine 3(2): 171-2.
31. Uhler, Kesen, Henderer, Steinmann (2002) Glaucoma screening at community senior centers: Follow-up assessment. INVESTIGATIVE OPHTHALMOLOGY & VISUAL SCIENCE Annual Meeting of the Association for Research in Vision and Ophthalmology MAY 05-10, 2002 FT LAUDERDALE, FLORIDA Assoc Res Vis & Ophthalmol. 43SU 2MA 3327.
32. van Zyl , Tave Annamey (2013) Prescheduled appointments as a strategy to improve follow-up rates among at-risk individuals identified during community-based glaucoma screenings. Yale University.
33. Ventura, Lemus, Parrish, Porciatti (2002) Screening for glaucoma with a simplified version of the pattern ERG called PERGLA: 49% of glaucoma suspects with abnormal disk and normal IOP and visual field have an abnormal response. INVESTIGATIVE OPHTHALMOLOGY & VISUAL SCIENCE Annual Meeting of the Association for Research in Vision and Ophthalmology MAY 05-10, 2002 FT LAUDERDALE, FLORIDASP Assoc Res Vis & Ophthalmol. 43SU 1MA 306.
34. Vistamehr, Shelsta, Palmisano, Filardo, Bashfor (2006) Glaucoma screening in a high-risk population. JOURNAL OF GLAUCOMA 15(6).
